# Process calculi may reveal the equivalence lying at the heart of RNA and proteins

**DOI:** 10.1038/s41598-018-36965-1

**Published:** 2019-01-24

**Authors:** Stefano Maestri, Emanuela Merelli

**Affiliations:** School of Science and Technology, University of Camerino, Camerino, 62032 Italy

**Keywords:** Computational models, Computational science

## Abstract

The successful use of process calculi to specify behavioural models allows us to compare RNA and protein folding processes from a new perspective. We model the folding processes as behaviours resulting from the interactions that nucleotides and amino acids (the elementary units that compose RNAs and proteins respectively) perform on their linear sequences. This approach is intended to provide new knowledge about the studied systems without strictly relying on empirical data. By applying Milner’s CCS process algebra to highlight the distinguishing features of the two folding processes, we discovered an abstraction level at which they show behavioural equivalences. We believe that this result could be interpreted as a clue in favour of the highly-debated RNA World theory, according to which, in the early stages of cell evolution, RNA molecules played most of the functional and structural roles carried out today by proteins.

## Introduction

RNAs (ribonucleic acids) and proteins are two classes of molecules that have drawn the interest of different scientific disciplines due to the fundamental roles they play in many biological processes. The study of their folding processes represents an important issue to discover the qualitative information underlying the relation between their structures and functions.

They perform a similar pathway from their linear sequence to a three-dimensional conformation, which in turn allows them to carry out almost the same functions (i.e. catalytic and structural roles). Investigating the reasons of existence of such similar molecules leads to the formulation of the RNA World hypothesis: RNA might be a “fossil” of an RNA world, existed on Earth before modern cells appeared, in which RNA fulfilled the roles of both DNA and proteins. This theory is still highly debated^1,2^; indeed, beyond their similarities, proteins and RNAs show profound structural differences, which affect the way they perform their functions.

This article is intended to provide a formal description of the folding process of proteins compared to the one of RNAs; our purpose is to identify, by highlighting their key properties, clues of the validity of the RNA World hypothesis. We focus our study on the interactions carried out by the elementary units that compose RNAs and proteins (on their respective linear sequences), describing the whole folding process as the resulting behaviour of such interactions.

The definition of the models we propose in this paper is based on the idea that all the components involved in a system, and the communication media themselves, can be formally modelled as processes. This approach has been applied to study biological systems by modelling entire molecules^3,4^, and can be extended to analyse their substructures or even their elementary units, since it allows describing every kind of interaction they perform; it is also possible to identify similarities among different classes of molecules and in the functions they carry out.

The specification language that better suits our modelling of RNA and protein folding is the process algebra called CCS (Calculus of Communicating Systems), proposed by Milner in 1989^5^; thanks to this language it has been possible to define the congruence of the folding processes in terms of *behavioural equivalence* and also to perform the model checking with the aid of automated tools.

## Results

Before introducing our models of RNA and protein folding, we propose few fundamentals on process algebras necessary to understand our approach; more details about its application to reactive systems can be found in the book of Aceto *et al*.^6^ and in the following Section Methods.

Process algebras are prototype specification languages that consist of a collection of operations for building a new process description from existing ones. In this context, processes can be viewed as systems that exhibit a behaviour and interact via synchronised communication. In Milner’s CCS process algebra, a process is thought as a black box with a name and a set of communication channels. An output or input action on the channel *a* is indicated using the labels $\overline{a}$ or *a* respectively.

In our models we use the following process constructors. Let *P*, *Q* be processes:

**action prefixing:** if *a* is an action, *a*.*P* is a process that begins by performing the action *a* and behaves like *P* thereafter;

**choice operator:** *P* + *Q* is a process that may behave like *P* or *Q*;

**parallel composition:** *P*|*Q* describes a system in which *P* and *Q* run in parallel, proceeding independently or communicating via complementary channels;

**restriction:** if *L* is a set of channel names, then *P*\*L* is a process in which the scope of the channel names in *L* is restricted to *P*; this means that those channel names can only be used for communication within *P*.

The whole folding process has been modelled as the result of sub-processes that proceed along a path made by discrete states; this aspect has been highlighted by describing all the modelled processes via Labelled Transition Systems (LTSs)^7^; they consist of a set of processes, a set of actions and a transition relation → such that, if a process *P* can perform an action *a* and become a process *P*′, we write $P\,\mathop{\longrightarrow }\limits^{a}\,P^{\prime}$^6^.

We want to point out that some aspects contributing to the folding process that can be considered relevant from a biological point of view, like the role of helping molecules (e.g. the modulation performed by Mg^2+^ on the RNA folding or the action of molecular chaperones in protein folding^8,9^), have not been taken into account in our model. This choice has been driven by the idea of describing the folding process as a behaviour strictly resulting from the peculiar properties of the interactions carried out by nucleotides and amino acids (in their respective linear sequences) and of the informational content brought along by each of them.

If on the one hand such approach led us to define an abstraction of the actual folding mechanisms, on the other it allowed us to formally prove the existence of distinguishing features of these processes that might be the basis of the very existence of both RNAs and proteins in cells. We wanted to prove that the inner potentiality of each elementary unit to interact with the others (in the same sequence) is the main property that determines the different complexity eventually reachable by the two classes of molecules.

To demonstrate such statement, we started by defining the models of the two folding processes as a sequence of folding steps, each contributing with a new weak interaction between two units of the linear sequence of the molecule. In order for a folding step to take place, the weak interaction must cause a reduction in the free energy of the system.

Because the folding process relies mainly in the formation of weak, noncovalent interactions in both RNAs and proteins, the stabilising function performed by covalent bonds (like the disulphide bridges between Cys residues) can be considered negligible for the purpose of our modelisation.

Even if the weak interactions taken into account are the same for RNAs and proteins, the rules that allow two nucleotides to interact are different from the rules that determine the interplay of two amino acids; we modelled such rules starting from the biochemical properties of the weak interactions. Hence, we needed to define two different models, one for each class of molecules.

The differences highlighted affect the whole folding process and led our models to show different traces, which means different sequences of transitions in their respective LTSs.

However, the expressiveness of the modelling approach based on process algebras allowed us to identify an abstraction level in which these two processes show a congruence relation called *strong bisimilarity*. This means that they afford the same traces and that all the states they reach in such traces are equivalent^6^.

At this specific level of abstraction, the two folding processes lead to the formation of structures with the same complexity and hence capable to express the same functions.

If the same abstraction level might represent the actual folding process of RNAs and proteins, there would be no reasons for the existence of both these two classes of molecules in cell, showing the same behaviour. Conversely, according to the RNA World hypothesis, the fact that such similar molecules can still be found in nature, allows us to hypothesise that, in the early stages of cell evolution, RNA might be the only type of molecule that performed structural and catalytic activities; as the complexity of cells increased, also emerged the necessity of molecules able to carry out more complex tasks. Towards the RNA World hypothesis, these molecules (proteins) might be evolved on the same property that was characterising RNAs of being a linear sequence of elementary units able to fold up to a three-dimensional structure, driven by the free energy reduction. As we show with our models, the cells cope with this necessity by the formation of molecules whose elementary units (the amino acids) are able to perform more complex interactions than nucleotides. Our results concern the RNA World hypothesis due to the interpretation of the behavioral equivalence of RNA and protein folding under specific restrictions (as in Theorem 1).

In the models of the folding process that we have defined, the weak interactions are classified in three main categories:

- hydrogen bonds;
- electrostatic interactions (ionic and van der Waals);
- hydrophobic interactions.

The hydrogen bond can be defined as an electrostatic interaction, but, due to its distinctive properties and the fundamental role it carries out in the folding process, it has been represented separately. Moreover, the model of each weak interaction has to be contextualised in the folding step it belongs to.

### Folding step

A folding step represents an iteration that allows the non-deterministic choice between one of the possible sub-processes describing the behaviour of the weak-interactions.

A *Folding Step* process (${ {\mathcal F} }^{{\rm{s}}}$) ensures that each sub-process complies with the specific restrictions on its input (according to the descriptions given below in this document) and that the interaction has a negative *free-energy change*, ΔG, which measures the amount of disorder created in a system when an interaction takes place. It can assume the value negative (ndg), positive (pdg) or zero (zdg). An interaction is *energetically favorable* if it creates disorder by decreasing the free energy of the system, namely if it has a negative ΔG; this condition is essential for an interaction to be carried out.

In order to meet the last requirement, both the *RNA Folding Step* (${ {\mathcal F} }_{{\rm{rna}}}^{{\rm{s}}}$) and *Protein Folding Step* (${ {\mathcal F} }_{{\rm{p}}}^{{\rm{s}}}$) processes are placed in parallel composition with the process ${\Delta }{G}_{{ {\mathcal F} }^{{\rm{s}}}}$, which represents the ΔG variation during folding. In this way the whole folding processes, ${{\mathscr{F}}}_{{\rm{r}}{\rm{n}}{\rm{a}}}$ and ${ {\mathcal F} }_{{\rm{p}}}$ respectively, can be defined as following:

$$\begin{array}{lll}{ {\mathcal F} }_{{\rm{rna}}} & \mathop{=}\limits^{{\rm{def}}} & ({ {\mathcal F} }_{{\rm{rna}}}^{{\rm{s}}}|{\Delta }{G}_{{ {\mathcal F} }^{{\rm{s}}}})\backslash \{{\mathtt{ndg}},{\mathtt{pdg}},{\mathtt{zdg}}\};\\ { {\mathcal F} }_{{\rm{p}}} & \mathop{=}\limits^{{\rm{def}}} & ({ {\mathcal F} }_{{\rm{p}}}^{{\rm{s}}}|{\Delta }{G}_{{ {\mathcal F} }^{{\rm{s}}}})\backslash \{{\mathtt{ndg}},{\mathtt{pdg}},{\mathtt{zdg}}\};\end{array}$$

where

$$\Delta {G}_{{ {\mathcal F} }^{{\rm{s}}}}\mathop{=}\limits^{{\rm{def}}}\overline{{\mathtt{pdg}}}\mathrm{.}{\Delta }{G}_{{ {\mathcal F} }^{{\rm{s}}}}+\overline{{\mathtt{ndg}}}\mathrm{.}{\Delta }{G}_{{ {\mathcal F} }^{{\rm{s}}}}+\overline{{\mathtt{zdg}}}\mathrm{.}{\Delta }{G}_{{ {\mathcal F} }^{{\rm{s}}}}.$$

**Figure 1:**
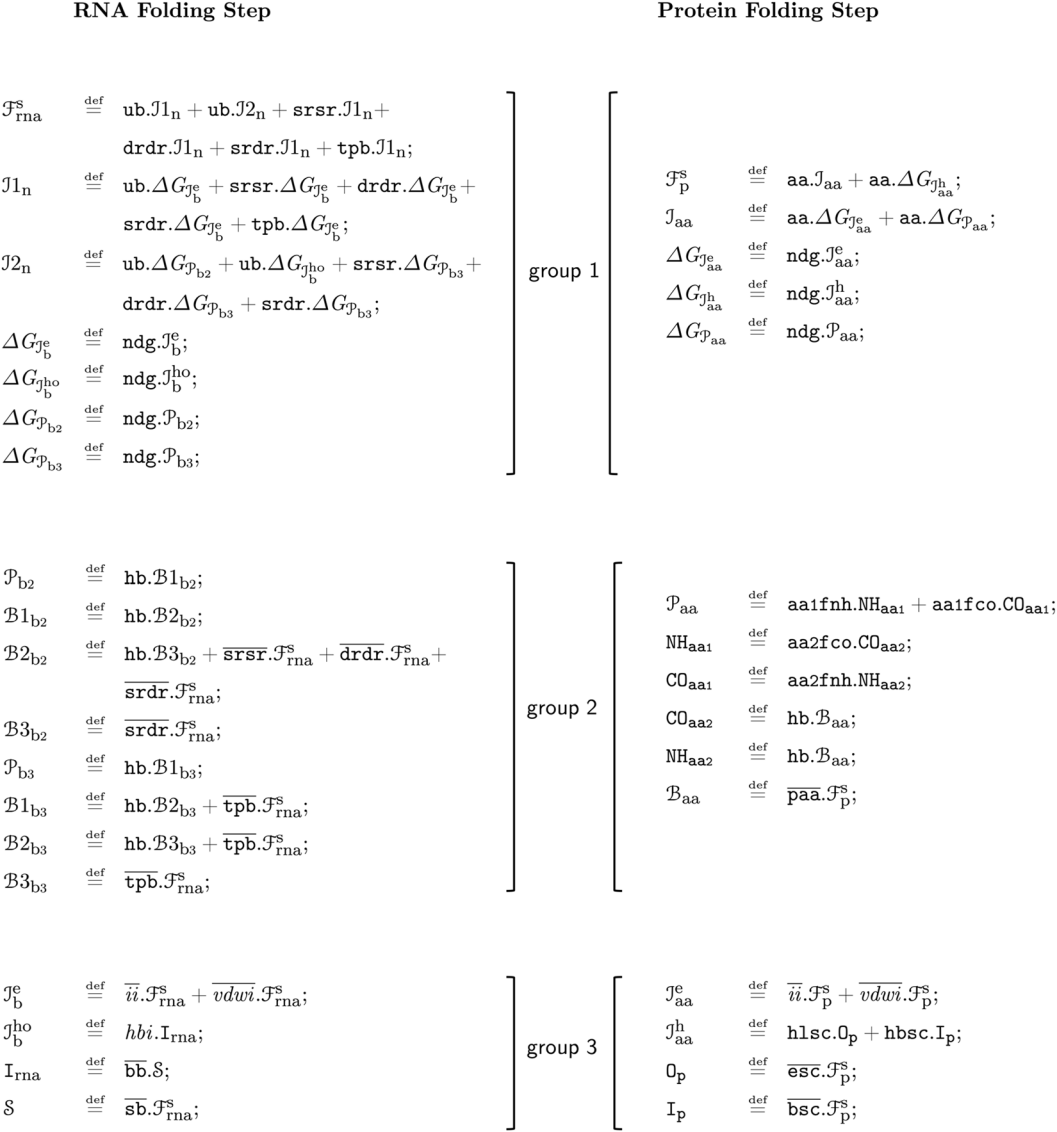
In this figure a comparative representation of the two folding step models (RNA on the left side and protein on the right) is proposed. Each model can be ideally divided into three groups of sub-processes; they have the function of determining the type of interacting elementary units and the interaction that is going to bind them (*group 1*), modelling the formation of hydrogen bonds (*group 2*) and of ionic and van der Waals interactions (*group 3*). For detailed information on the construction of the models and on the meaning of the symbols used, see Section 1 and 2 of the Supplementary Information.

Both ${ {\mathcal F} }_{{\rm{rna}}}^{{\rm{s}}}$ and ${ {\mathcal F} }_{{\rm{p}}}^{{\rm{s}}}$ are structured in sub-processes that can be clustered in three main groups (see Fig. 1):

*group 1* determines the type of the elementary units involved in the ongoing folding step, the interaction that is going to establish between them and if its ΔG is negative;

*group 2* describes the formation of one or more hydrogen bonds between two units (unpaired or already paired);

*group 3* models the behaviour of ionic, van der Waals and hydrophobic interactions.

In this first phase of our modelisation, which aims to remain as faithful as possible to the biological folding process, the *group 2* of sub-processes carries out the important task of limiting the maximum number of elementary units that can be linked by hydrogen bonds as well as the number of hydrogen bonds that can be generated between two units.

The hydrogen bond formation (in both Watson-Crick and Wobble base pair) has been modelled generalising this process as an interaction between a purine (adenine or guanine - labelled dr, since they are **d**ouble-**r**ing bases) and a pyrimidine (uracil and cytosine - **s**ingle-**r**ing bases and hence labelled sr) or between a two paired bases and a third base (also in this case, a generic purine or pyrimidine). The base pairing is symmetric, thus: srdr = drsr.

Regarding the number of hydrogen bonds allowed in a base pair, in our models they must be at least two and at most three; the number of hydrogen bonds that link an unpaired base to a group of two already paired bases must be from one to three. It has been decided to limit the minimum number of hydrogen bonds in a base pair (to the number of two) because base pairs with a single hydrogen bond can be classified as a variant of the primary types and because the whole number of hydrogen bonds found in a base triplet is at least three^10^.

In contrast with the base pairing of nucleotides, only a single hydrogen bond is allowed between two amino acids; however, there is no limitation in the length of a sequence of amino acids linked to one another via hydrogen bonds.

A complete description of the conventions adopted and the choices made to derive the two models from the biological folding processes can be found in the Supplementary Information, whose Section 1 explains the symbols used in the models and their transliteration while Section 2 the models construction).

### Bisimilarity equivalence

The verification that two processes of the proposed models are bisimilar (i.e. if they show the same behaviour) is based on *bisimulation games*, namely game characterizations of the bisimilarity. Informally, we can define a bisimulation game as a sequence of rounds in which the LTSs of two processes are compared. The game explores the LTSs by pairs of states (called configurations).

Starting from an initial configuration, two players, an attacker and a defender, try to perform in turn a transition basing on one of the two LTSs; the game is begun by the attacker, who decides which transition of the initial configuration to perform (and hence which of the two LTSs to explore). The choice made in each turn determines the configuration explored in the next one by the other player. A finite play of the game is lost by the player who cannot make a move from the current configuration. If the play is infinite (as in the case in which a cycle is detected) the game is considered won by the defender (because the attacker is unable to distinguish the behaviour of the two processes).

Two states are strongly bisimilar if and only if the defender has a *universal winning strategy* (i.e., he can always win the game, regardless of how the attacker selects his moves) in the strong bisimulation game that starts from the configuration made by such states.

**Figure 2:**
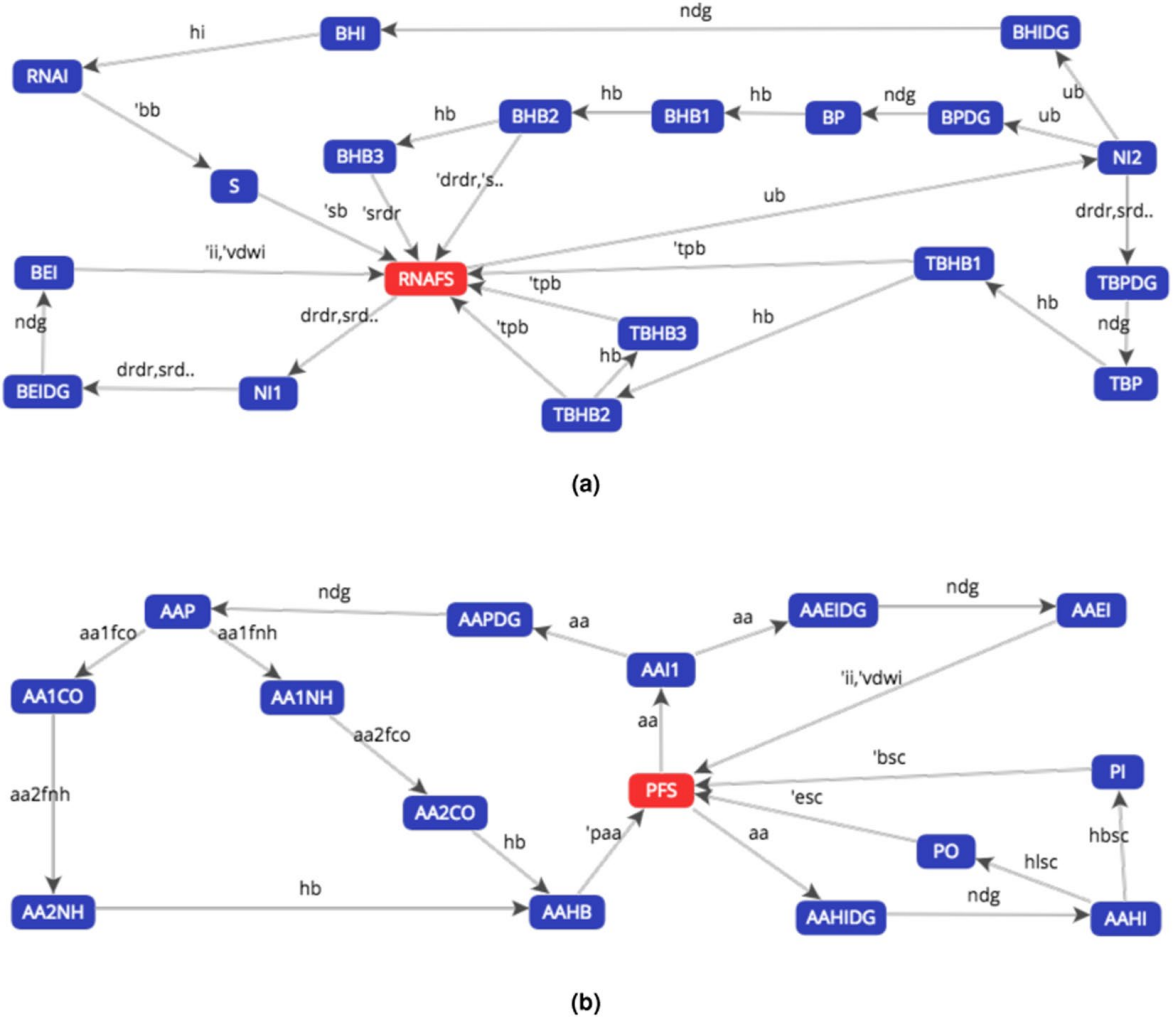
Labelled Transition Systems of **(a)** the ${ {\mathcal F} }_{{\rm{rna}}}^{{\rm{s}}}$ process, transliterated RNAFS, and of **(b)** the ${ {\mathcal F} }_{{\rm{p}}}^{{\rm{s}}}$ process, transliterated PFS, generated with the CAAL web-based tool (Concurrency Workbench, Alborg Edition). The symbols are described in Section 1 of the Supplementary Information.

If we try to prove the behavioural equivalence of the ${ {\mathcal F} }_{{\rm{rna}}}^{{\rm{s}}}$ and ${ {\mathcal F} }_{{\rm{p}}}^{{\rm{s}}}$ processes we can observe, from the LTSs in Fig. 2, that the bisimulation game ends after only one move, independently of the choice made by the attacker, with the defeat of the defender.

As an example, if the attacker chooses the transition ${\rm{R}}{\rm{N}}{\rm{A}}{\rm{F}}{\rm{S}}\,\mathop{\longrightarrow }\limits^{{\rm{u}}{\rm{b}}}\,{\rm{N}}{\rm{I}}2$ on the RNAFS LTS, the defender has no available transition on the PFS LTS to respond.

This first verification proves that a model strictly faithful to the biological folding leads us to define processes whose behaviours are not equivalent.

We might therefore wonder if *there is an abstraction level at which the two folding processes would show a behavioural equivalence*. As it will be proved in this article, this level of abstraction can actually be defined. Its construction, however, requires a generalisation of the weak-interaction processes and the imposition of some limitations to the expressiveness of the protein folding process.

### Higher abstraction level model

The first of the two aforementioned modifications can be achieved by:

- redefining nucleotides and the amino acids as general elementary units, which can be paired or unpaired;
- abstracting from the specificity of each pairing process by no longer taking into account the number of hydrogen bonds formed between two (or three) paired units;
- generalising the hydrophobic interactions to their key feature of burying the hydrophobic molecules while exposing the hydrophilic ones (no longer considering the stacking process typical of the hydrophobic interactions of nucleotides).

These adjustments to the model do not affect the main property of each weak interaction, therefore the model is still faithful to the biological process. However, they are not sufficient to obtain a behavioural equivalence between the folding processes of RNAs and proteins.

What we still need to do is limiting the folding capability of the proteins by reducing the number of amino acids that can interact through hydrogen bonds to the number of three (the maximum number of nucleotides that can pair in RNAs).

Let ${\mathcal H} :P\to P$ be the function that maps each folding process to its respective abstraction level, as above defined. The application of ${\mathcal H}$ to the models described in the previous section results in a new representation of the folding processes of RNAs and proteins, indicated by the symbols ${{\mathscr{F}}}_{{\rm{r}}{\rm{n}}{\rm{a}}}$ and ${{\mathscr{F}}}_{{\rm{p}}}$ respectively (see Section 2 of the Supplementary Information for a complete description).

The definition of these new models can be considered an important result since it is possible to prove that, at this level of abstraction, the RNA folding process and the protein folding process show the same behaviour.

#### **Theorem 1**.

*If* ${{\mathscr{F}}}_{{\rm{r}}{\rm{n}}{\rm{a}}}={\mathscr{H}}({{\mathscr{F}}}_{{\rm{r}}{\rm{n}}{\rm{a}}})$ *and* ${{\mathscr{F}}}_{{\rm{p}}}={\mathscr{H}}({{\mathscr{F}}}_{{\rm{p}}})$ then ${{\mathscr{F}}}_{{\rm{r}}{\rm{n}}{\rm{a}}}$ and ${{\mathscr{F}}}_{{\rm{p}}}$ are strongly bisimilar (${{\mathscr{F}}}_{{\rm{r}}{\rm{n}}{\rm{a}}}\sim {{\mathscr{F}}}_{{\rm{p}}}$).

#### *Proof*.

**Figure 3:**
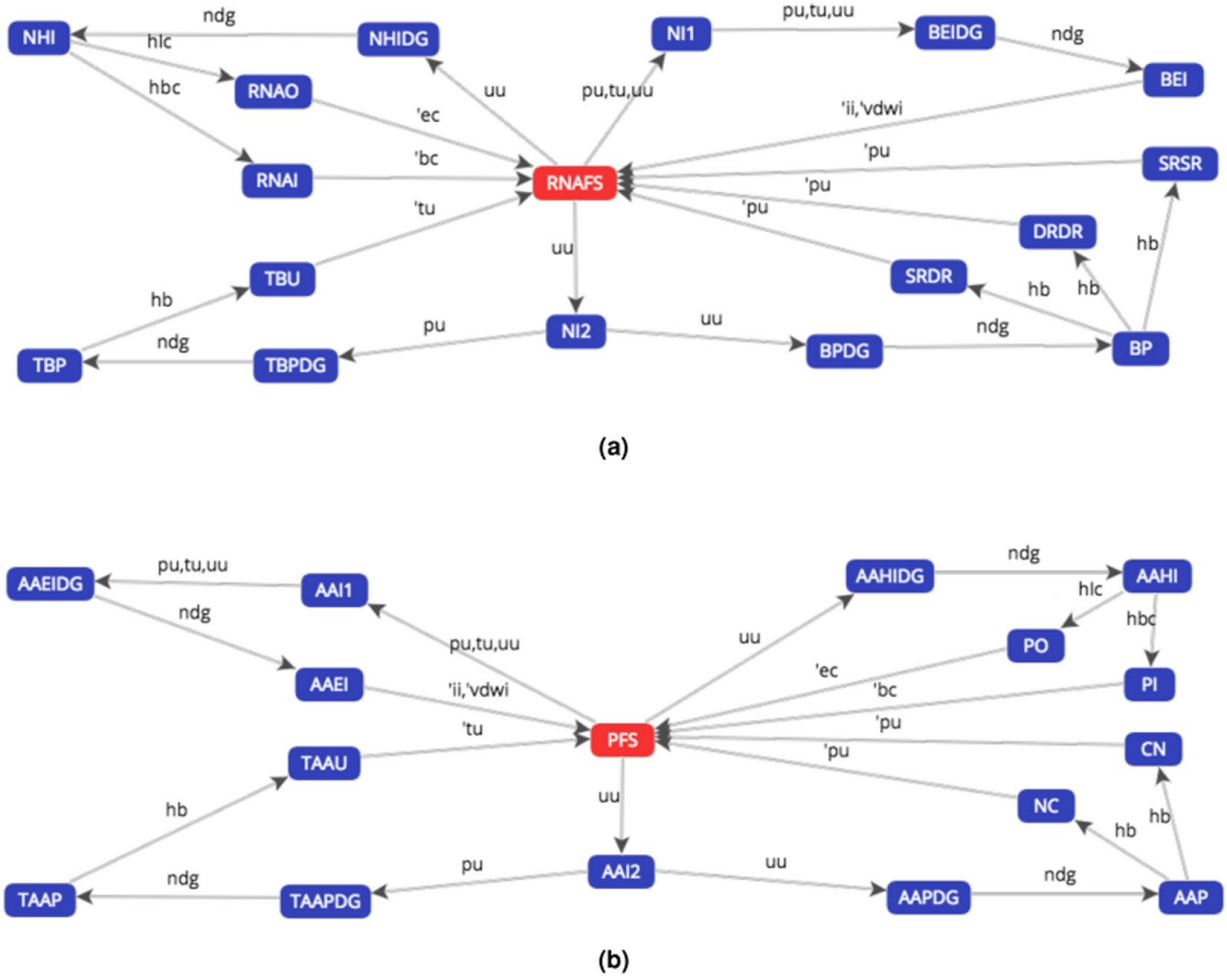
Labelled Transition Systems of **(a)** the redefined ${{\mathscr{F}}}_{{\rm{r}}{\rm{n}}{\rm{a}}}^{{\rm{s}}}$ process, transliterated RNAFS, and of **(b)** the redefined ${{\mathscr{F}}}_{{\rm{p}}}^{{\rm{s}}}$ process, transliterated PFS, generated with the CAAL web-based tool (Concurrency Workbench, Alborg Edition). The symbols are described in Section 1 of the Supplementary Information.

The proof is provided via a bisimulation game (see Table 1). A winning strategy of the defender starts from the pair of states (${{\mathscr{F}}}_{{\rm{r}}{\rm{n}}{\rm{a}}}^{{\rm{s}}},{{\mathscr{F}}}_{{\rm{p}}}^{{\rm{s}}}$) of the relative LTSs, transliterated (RNAFS, PFS) as in Fig. 3.

**Table 1:** Winning strategy of the defender in the strong bisimulation game that compares the pair of processes (${{\mathscr{F}}}_{{\rm{r}}{\rm{n}}{\rm{a}}}^{{\rm{s}}},{{\mathscr{F}}}_{{\rm{p}}}^{{\rm{s}}}$), transliterated (RNAFS, PFS). The results of this play proves that RNAFS ~ PFS, i.e. that the two processes are strongly bisimilar.

Round	Current configuration	Attacker	Defender
Round 1	(RNAFS, PFS)	${\rm{RNAFS}}\,\mathop{\longrightarrow }\limits^{{\rm{uu}}}\,{\rm{NI}}2$	${\rm{PFS}}\,\mathop{\longrightarrow }\limits^{{\rm{uu}}}\,{\rm{AAI}}2$
Round 2	(NI2, AAI2)	${\rm{NI}}2\,\mathop{\longrightarrow }\limits^{{\rm{uu}}}\,{\rm{BPDG}}$	${\rm{AAI}}2\,\mathop{\longrightarrow }\limits^{{\rm{uu}}}\,{\rm{AAPDG}}$
Round 3	(BPDG, AAPDG)	${\rm{BPDG}}\,\mathop{\longrightarrow }\limits^{{\rm{ndg}}}\,{\rm{BP}}$	${\rm{AAPDG}}\,\mathop{\longrightarrow }\limits^{{\rm{ndg}}}\,{\rm{AAP}}$
Round 4	(BP, AAP)	${\rm{BP}}\,\mathop{\longrightarrow }\limits^{{\rm{hb}}}\,{\rm{SRDR}}$	${\rm{AAP}}\,\mathop{\longrightarrow }\limits^{{\rm{hb}}}\,{\rm{CN}}$
Round 5	(SRDR, CN)	${\rm{SRDR}}\,\mathop{\longrightarrow }\limits^{\overline{{\rm{pu}}}}\,{\rm{RNAFS}}$	${\rm{CN}}\,\mathop{\longrightarrow }\limits^{{\rm{uu}}}\,{\rm{PFS}}$
Round 6	(RNAFS, PFS)	A cycle has been detected	Defender wins

As proved by Milner^5^, given two processes *P* and *Q*, such that *P* ~ *Q*, the following two rules are true:

$P|R\sim Q|R$ and $R|P\sim R|Q$, for each process *R*

$P\backslash L\sim Q\backslash L$, for each set of labels *L*,

The ${{\mathscr{F}}}_{{\rm{r}}{\rm{n}}{\rm{a}}}$ and ${{\mathscr{F}}}_{{\rm{p}}}$ folding processes, likewise ${ {\mathcal F} }_{{\rm{rna}}}$ and ${ {\mathcal F} }_{{\rm{p}}}$, are defined as

$$\begin{array}{ccc}{{\mathscr{F}}}_{{\rm{r}}{\rm{n}}{\rm{a}}} & \mathop{=}\limits^{{\rm{d}}{\rm{e}}{\rm{f}}} & ({{\mathscr{F}}}_{{\rm{r}}{\rm{n}}{\rm{a}}}^{{\rm{s}}}|{\rm{\Delta }}{G}_{{{\mathscr{F}}}^{{\rm{s}}}}){\rm{\setminus }}\{{\mathtt{n}}{\mathtt{d}}{\mathtt{g}},{\mathtt{p}}{\mathtt{d}}{\mathtt{g}},{\mathtt{z}}{\mathtt{d}}{\mathtt{g}}\};\\ {{\mathscr{F}}}_{{\rm{p}}} & \mathop{=}\limits^{{\rm{d}}{\rm{e}}{\rm{f}}} & ({{\mathscr{F}}}_{{\rm{p}}}^{{\rm{s}}}|{\rm{\Delta }}{G}_{{{\mathscr{F}}}^{{\rm{s}}}}){\rm{\setminus }}\{{\mathtt{n}}{\mathtt{d}}{\mathtt{g}},{\mathtt{p}}{\mathtt{d}}{\mathtt{g}},{\mathtt{z}}{\mathtt{d}}{\mathtt{g}}\};\end{array}$$

where

$${\rm{\Delta }}{G}_{{{\mathscr{F}}}^{{\rm{s}}}}\mathop{=}\limits^{{\rm{d}}{\rm{e}}{\rm{f}}}\,\bar{{\mathtt{p}}{\mathtt{d}}{\mathtt{g}}}.{\rm{\Delta }}{G}_{{{\mathscr{F}}}^{{\rm{s}}}}+\bar{{\mathtt{n}}{\mathtt{d}}{\mathtt{g}}}.{\rm{\Delta }}{G}_{{{\mathscr{F}}}^{{\rm{s}}}}+\bar{{\mathtt{z}}{\mathtt{d}}{\mathtt{g}}}.{\rm{\Delta }}{G}_{{{\mathscr{F}}}^{{\rm{s}}}}.$$

Then they are also strongly bisimilar.

In this way we have formally demonstrated the existence of an abstraction level at which the folding processes of RNAs and proteins show the same behaviour and hence can generate three-dimensional structures of the same complexity.

**Figure 4:**
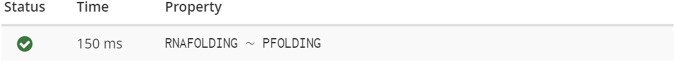
Bisimulation game performed with the CAAL web-based tool shows that, as the checkmark on the “Status” column indicates, the RNAFOLDING and the PFOLDING processes are strongly bisimilar (relation represented by the symbol ~).

Such proof can also be obtained with the aid of an automated tool; in Fig. 4 we show the results of the bisimulation game performed with CAAL on the processes ${{\mathscr{F}}}_{{\rm{r}}{\rm{n}}{\rm{a}}}$ and ${{\mathscr{F}}}_{{\rm{p}}}$, transliterated RNAFOLDING and PFOLDING respectively.

## Discussion

Starting from the models of RNA and protein folding, we have demonstrated how it is possible to formally define an abstraction level at which such processes show a behavioural equivalence. Its existence allows us to hypothesise some of the reasons that led the evolution of life to the formation of the proteins and to take them on, in biological processes, along with RNAs.

We have formally proved how it is possible to reach the behavioural equivalence between the RNA folding and the protein folding by reducing the complexity of the structures expressible, hence the functions they can perform, in the latter process. This demonstration can be interpreted as a clue that, at a point in the early evolution of life on Earth, proteins emerged to answer the necessity of molecules that could carry out more effectively the functions performed by RNA molecules and could also deal with more complex tasks. We are well aware that this demonstration leaves numerous questions open regarding the RNA World theory, such as the function that RNA would play in storing genetic information; it is not in any case the objective of our work to provide a definitive proof of the aforementioned theory. However, we are equally convinced that our work sets a solid foundation for further developments in this direction.

Indeed, thanks to these results, we can observe how it is possible to infer the complexity of a biological structure, and therefore of its function, starting from the properties of its elementary components. In the case of RNAs and proteins, the distinguishing features of their respective folding processes have been identified and modelled only on the basis of the known properties of the interactions that bind nucleotides (in RNAs) and amino acids (in proteins).

CCS, due to its expressiveness, turned out to be perfectly suitable to define models based on the application of the aforementioned approach. The use of process algebras to describe molecular interactions can highlight the relation between the complexity of the functions carried out by a biological entity and the type of interactions tying the elementary units that compose its structure.

This idea could be extended to the definition of predictive models of many other classes of biological molecules and processes, by taking into account all the fundamental dynamics characterising a biological system. We are currently involved in defining formal models of the whole gene expression process in order to study the gene mutations which cause protein misfolding^11,12^ and the gene assembly process^13^.

Our approach should not be intended as a simulation-based tool, but a theoretical way to acquire new knowledge about the studied systems. However, we have not aimed to define a new theory, but a new methodology to understand biological behaviours by analysing the complexity of the interactions characterising living systems. Moreover, our work can be placed in the context of the topological analysis of the folding process^14–16^.

Although the results proposed in the present article are based on the construction of algebraic models through process calculi, they actually provide us with factual knowledge. We believe that mathematics is not about human activity or phenomena, it is about the extraction and formalization of ideas and their manifold consequences^17^.

## Methods

This section presents an essential description of the concepts at the basis of the models proposed in this article. The description is mainly based on the book of Aceto *et al*.^6^.

### Labelled Transition Systems

A labelled transition system (LTS) is a triple $({\bf{Proc}},{\bf{Act}},\{\mathop{\longrightarrow }\limits^{a}|\,a\in {\bf{Act}}\})$, where:

- **Proc** is a set of states (or processes);
- **Act** is a set of actions (or labels);
- $\mathop{\longrightarrow }\limits^{a}\subseteq {\bf{Proc}}\times {\bf{Proc}}$ is a transition relation, for every *a* ∈ **Act**.

**CCS syntax**

 $$A\,\text{Set of channel names}$$

 $$\bar{A}=\{\bar{a}|a\in A\}\,\mathrm{Set}\,\mathrm{of}\,\mathrm{complementary}\,\mathrm{names}$$

 $${\mathscr{L}}=A\cup \bar{A}\,\text{Set of labels}$$

 $${\bf{A}}{\bf{c}}{\bf{t}}={\mathscr{L}}\cup \{\tau \}\,\text{Set of actions, where}\,\tau \,\text{is an unobservable action}$$

 $${\mathscr{K}}\,\text{Set of process names (constants)}$$

The set ${\mathscr{P}}$ of the CCS expression, is given by the following grammar:

$$P,Q\,:\,:\,=K\,|\,\alpha .\,P\,|\,\sum _{i\in I}{P}_{i}\,|\,P|Q\,|\,P[f]\,|\,P\backslash L$$

Where:

- *K* is a process name in ${\mathscr{K}}$;
- *α* is an action in **Act**;
- *I* is a possibly infinite index set;
- $f:{\bf{Act}}\to {\bf{Act}}$ is a relabelling function satisfying the following constraints:
  - $$f({\rm{\tau }})={\rm{\tau }}$$
  - $f(\bar{a})=\overline{f(a)}$ for each label *a*;
- *L* is a set of labels from ${\mathcal L}$.

The behaviour of each process constant *K* $\in {\mathscr{K}}$ is given by a defining equation $K\,\mathop{=}\limits^{{\rm{def}}}P$, where $P\in {\mathscr{P}}$.

**CCS Structural Operational Semantics**

α ∈ **Act** and $$a\in  {\mathcal L} $$,

$$\begin{array}{cc}\bar{\alpha .P\,\mathop{\longrightarrow }\limits^{\alpha }\,P} & \text{Action prefixing}\\ \frac{{P}_{j}\,\mathop{\longrightarrow }\limits^{\alpha }\,{P}_{j}^{{\rm{^{\prime} }}}}{\sum _{i\in I}{P}_{i}\,\mathop{\longrightarrow }\limits^{\alpha }\,{P}_{j}^{{\rm{^{\prime} }}}}\,{\rm{w}}{\rm{h}}{\rm{e}}{\rm{r}}{\rm{e}}\,j\in I & \text{Summation}\\ \frac{P\,\mathop{\longrightarrow }\limits^{\alpha }\,{P}^{{\rm{^{\prime} }}}}{P|Q\,\mathop{\longrightarrow }\limits^{\alpha }\,{P}^{{\rm{^{\prime} }}}|Q} & \text{Parallel composition (rule 1)}\\ \frac{Q\,\mathop{\longrightarrow }\limits^{\alpha }\,{Q}^{{\rm{^{\prime} }}}}{P|Q\,\mathop{\longrightarrow }\limits^{\alpha }\,P|{Q}^{{\rm{^{\prime} }}}} & \text{Parallel composition (rule 2)}\\ \frac{P\,\mathop{\longrightarrow }\limits^{a}\,{P}^{{\rm{^{\prime} }}}\,Q\mathop{\longrightarrow }\limits^{\bar{a}}{Q}^{{\rm{^{\prime} }}}}{P|Q\,\mathop{\longrightarrow }\limits^{\tau }\,{P}^{{\rm{^{\prime} }}}|{Q}^{{\rm{^{\prime} }}}} & \text{Parallel composition (rule 3)}\\ \frac{P\,\mathop{\longrightarrow }\limits^{\alpha }\,{P}^{{\rm{^{\prime} }}}}{P{\rm{\setminus }}L\,\mathop{\longrightarrow }\limits^{\alpha }\,{P}^{{\rm{^{\prime} }}}{\rm{\setminus }}L}\,{\rm{w}}{\rm{h}}{\rm{e}}{\rm{r}}{\rm{e}}\,\alpha \notin L & \text{Restriction}\\ \frac{P\,\mathop{\longrightarrow }\limits^{\alpha }\,{P}^{{\rm{^{\prime} }}}}{P[f]\,\mathop{\longrightarrow }\limits^{f(\alpha )}\,{P}^{{\rm{^{\prime} }}}[f]} & \text{Relabelling}\\ \frac{P\,\mathop{\longrightarrow }\limits^{\alpha }\,{P}^{{\rm{^{\prime} }}}}{K\,\mathop{\longrightarrow }\limits^{\alpha }\,{P}^{{\rm{^{\prime} }}}}\,{\rm{w}}{\rm{h}}{\rm{e}}{\rm{r}}{\rm{e}}\,K\mathop{=}\limits^{{\rm{d}}{\rm{e}}{\rm{f}}}P & \text{Constant definition}\end{array}$$

### Strong bisimulation

A binary relation ${\mathcal R}$ over the set of states of an LTS is a bisimulation iff whenever ${s}_{1}\, {\mathcal R} \,{s}_{2}$ and *α* is an action:

- if ${s}_{1}\mathop{\longrightarrow }\limits^{{\rm{\alpha }}}{s}_{1}^{\prime}$, then there is a transition ${s}_{2}\mathop{\longrightarrow }\limits^{{\rm{\alpha }}}{s}_{2}^{\prime}$ such that ${s}_{1}^{\prime} \, {\mathcal R} \,{s}_{2}^{\prime}$;
- if ${s}_{2}\mathop{\longrightarrow }\limits^{\alpha }{s}_{2}^{{\rm{^{\prime} }}}$, then there is a transition ${s}_{1}\mathop{\longrightarrow }\limits^{{\rm{\alpha }}}{s}_{1}^{\prime}$ such that ${s}_{1}^{\prime} \, {\mathcal R} \,{s}_{2}^{\prime}$.

Two states *s* and *s*′ are bisimilar, written *s* ~ *s*′, iff there is a bisimulation that relates them. The relation ~ will be referred to as strong bisimulation equivalence or strong bisimilarity.

## Supplementary information

Supplementary Information

## Data Availability

No datasets were generated or analysed during the current study.
