# Publisher Correction: Process calculi may reveal the equivalence lying at the heart of RNA and proteins

**DOI:** 10.1038/s41598-019-51082-3

**Published:** 2019-11-07

**Authors:** Stefano Maestri, Emanuela Merelli

**Affiliations:** School of Science and Technology, University of Camerino, Camerino, 62032 Italy

Correction to: *Scientific Reports* 10.1038/s41598-018-36965-1, published online 24 January 2019

This Article contains errors introduced during its publication, where the wrong fonts were used to distinguish between two models.

In the HTML version, in the Results section under subheading ‘Folding step’,

“In this way the whole folding processes, ${{\mathscr{{F}}}}_{rna}$ and ${ {\mathcal F} }_{p}$ respectively, can be defined as following”:

should read:

“In this way the whole folding processes, ${ {\mathcal F} }_{rna}$ and ${ {\mathcal F} }_{p}$ respectively, can be defined as following”:

In the same section of the HTML version, ‘Theorem 1’,

“If ${{\mathscr{{F}}}}_{rna}={\mathscr{{H}}}({{\mathscr{{F}}}}_{rna})$ *and* ${{\mathscr{{F}}}}_{p}={\mathscr{{H}}}({{\mathscr{{F}}}}_{p})$ then ${{\mathscr{{F}}}}_{rna}$ and ${{\mathscr{{F}}}}_{p}$ are strongly bisimilar (${{\mathscr{{F}}}}_{rna}\, \sim \,{{\mathscr{{F}}}}_{p}$)”.

should read:

“If ${{\mathscr{{F}}}}_{rna}= {\mathcal H} ({ {\mathcal F} }_{rna})$ *and* ${{\mathscr{{F}}}}_{p}= {\mathcal H} ({ {\mathcal F} }_{p})$ then ${{\mathscr{{F}}}}_{rna}$ and ${{\mathscr{{F}}}}_{p}$ are strongly bisimilar (${{\mathscr{{F}}}}_{rna}\, \sim \,{{\mathscr{{F}}}}_{p}$)”.

In the PDF version, in the Results section under subheading ‘Higher abstraction level model’,

“The application of ${\mathcal H}$ to the models described in the previous section results in a new representation of the folding processes of RNAs and proteins, indicated by the symbols ${ {\mathcal F} }_{rna}$ and ${ {\mathcal F} }_{p}$ respectively (see Section 2 of the Supplementary Information for a complete description)”.

should read:

“The application of ${\mathcal H}$ to the models described in the previous section results in a new representation of the folding processes of RNAs and proteins, indicated by the symbols ${{\mathscr{{F}}}}_{rna}$ and ${{\mathscr{{F}}}}_{p}$ respectively (see Section 2 of the Supplementary Information for a complete description)”.

In the same section of the PDF version, ‘Theorem 1’,

“If ${ {\mathcal F} }_{rna}= {\mathcal H} ({ {\mathcal F} }_{rna})$ *and* ${ {\mathcal F} }_{p}= {\mathcal H} ({ {\mathcal F} }_{p})$ then ${ {\mathcal F} }_{rna}$ and ${ {\mathcal F} }_{p}$ are strongly bisimilar (${ {\mathcal F} }_{rna}\, \sim \,{ {\mathcal F} }_{p}$)”.

should read:

“If ${{\mathscr{{F}}}}_{rna}= {\mathcal H} ({ {\mathcal F} }_{rna})$ *and* ${{\mathscr{{F}}}}_{p}= {\mathcal H} ({ {\mathcal F} }_{p})$ then ${{\mathscr{{F}}}}_{rna}$ and ${{\mathscr{{F}}}}_{p}$ are strongly bisimilar (${{\mathscr{{F}}}}_{rna}\, \sim \,{{\mathscr{{F}}}}_{p}$)”.

In the proof for Theorem 1,

“A winning strategy of the defender starts from the pair of states (${ {\mathcal F} }_{rna}^{s},{ {\mathcal F} }_{p}^{s}$) of the relative LTSs, transliterated (RNAFS, PFS) as in Fig. 3”.

should read:

“A winning strategy of the defender starts from the pair of states (${{\mathscr{{F}}}}_{rna}^{s},{{\mathscr{{F}}}}_{p}^{s}$) of the relative LTSs, transliterated (RNAFS, PFS) as in Fig. 3”.

In the same section of the PDF version,

“The ${ {\mathcal F} }_{rna}$ and ${ {\mathcal F} }_{p}$ folding processes, likewise ${ {\mathcal F} }_{rna}$ and ${ {\mathcal F} }_{p}$, are defined as

$${\mathcal F}_{rna}\mathop{=}\limits^{\rm{def}}({\mathcal F}_{rna}^{s}|\Delta {G}_{{\mathcal{F}}^{s}})\backslash \{ndg,\,pdg,\,zdg\};$$

$${\mathcal F}_{p}\mathop{=}\limits^{\rm{def}}({\mathcal F}_{p}^{s}|\Delta {G}_{{\mathcal{F}}^{s}})\backslash \{ndg,\,pdg,\,zdg\};$$

where

$$\Delta {G}_{{{\mathcal{{F}}}}^{s}}\mathop{=}\limits^{{\rm{def}}}\overline{pdg}.\,\Delta {G}_{{{\mathcal{{F}}}}^{s}}+\overline{ndg}.\,\Delta {G}_{{{\mathcal{{F}}}}^{s}}+\overline{zdg}.\,\Delta {G}_{{{\mathcal{{F}}}}^{s}}$$”

should read:

“The ${{\mathscr{{F}}}}_{rna}$ and ${{\mathscr{{F}}}}_{p}$ folding processes, likewise ${ {\mathcal F} }_{rna}$ and ${ {\mathcal F} }_{p}$, are defined as

$${{\mathscr{{F}}}}_{rna}\mathop{=}\limits^{{\rm{def}}}({{\mathscr{{F}}}}_{rna}^{s}|\Delta {G}_{{{\mathscr{{F}}}}^{s}})\backslash \{ndg,\,pdg,\,zdg\};$$

$${{\mathscr{{F}}}}_{p}\mathop{=}\limits^{{\rm{def}}}({{\mathscr{{F}}}}_{p}^{s}|\Delta {G}_{{{\mathscr{{F}}}}^{s}})\backslash \{ndg,\,pdg,\,zdg\};$$

where

$$\Delta {G}_{{{\mathscr{{F}}}}^{s}}\mathop{=}\limits^{{\rm{def}}}\overline{pdg}.\,\Delta {G}_{{{\mathscr{{F}}}}^{s}}+\overline{ndg}.\,\Delta {G}_{{{\mathscr{{F}}}}^{s}}+\overline{zdg}.\,\Delta {G}_{{{\mathscr{{F}}}}^{s}}$$”

And,

“Such proof can also be obtained with the aid of an automated tool; in Fig. 4 we show the results of the bisimulation game performed with CAAL on the processes ${ {\mathcal F} }_{rna}$ and ${ {\mathcal F} }_{p}$, transliterated RNAFOLDING and PFOLDING respectively”.

should read:

“Such proof can also be obtained with the aid of an automated tool; in Fig. 4 we show the results of the bisimulation game performed with CAAL on the processes ${{\mathscr{{F}}}}_{rna}$ and ${{\mathscr{{F}}}}_{p}$, transliterated RNAFOLDING and PFOLDING respectively”.

In the legend of Table 1 in the PDF version,

“Winning strategy of the defender in the strong bisimulation game that compares the pair of processes (${ {\mathcal F} }_{rna}^{s},{ {\mathcal F} }_{p}^{s}$), transliterated (RNAFS, PFS). The results of this play proves that RNAFS ~ PFS, i.e. that the two processes are strongly bisimilar”.

should read:

“Winning strategy of the defender in the strong bisimulation game that compares the pair of processes (${{\mathscr{{F}}}}_{rna}^{s},{{\mathscr{{F}}}}_{p}^{s}$), transliterated (RNAFS, PFS). The results of this play proves that RNAFS ~ PFS, i.e. that the two processes are strongly bisimilar”.

Finally, in the legend of Figure 3 in the PDF version,

“Labelled Transition Systems of (a) the redefined ${ {\mathcal F} }_{rna}^{s}$ process, transliterated RNAFS, and of (b) the redefined ${ {\mathcal F} }_{p}^{s}$, process, transliterated PFS, generated with the CAAL web-based tool (Concurrency Workbench, Alborg Edition)”.

should read:

“Labelled Transition Systems of (a) the redefined ${{\mathscr{{F}}}}_{rna}^{s}$ process, transliterated RNAFS, and of (b) the redefined ${{\mathscr{{F}}}}_{p}^{s}$, process, transliterated PFS, generated with the CAAL web-based tool (Concurrency Workbench, Alborg Edition)”.

