# Supplementary material for: Process calculi may reveal the equivalence lying at the heart of RNA and proteins
Source: Sci Rep. 2019 Jan 24;9:559. doi: 10.1038/s41598-018-36965-1 (PMC6345955; doi:10.1038/s41598-018-36965-1)
Supplement: Supplementary file 1 — Supplementary Information [file 41598_2018_36965_MOESM1_ESM.pdf]

Supplementary Information to *Process calculi may reveal the equivalence lying at the heart of RNA and proteins*

Stefano Maestri and Emanuela Merelli*

School of Science and Technology, University of Camerino, Camerino, 62032, Italy
*emanuela.merelli@unicam.it

1 Symbols and their transliteration

The following tables explain the symbols used to describe processes and actions of the proposed models; the transliterations of process names are necessary to construct the LTS representations as well as to perform the model checking and the bisimulation games through the automated tool CAAL (Concurrency Workbench, Alborg Edition).

Table 1.1: Processes

State\Process	Transliteration	Description
$\mathtt{NH}_{\mathtt{aa1}}$	AA1NH	first amino acid free amino group
$\mathtt{CO}_{\mathtt{aa1}}$	AA1CO	first amino acid free carboxyl group
$\mathtt{NH}_{\mathtt{aa2}}$	AA2NH	second amino acid free amino group
$\mathtt{CO}_{\mathtt{aa2}}$	AA2CO	second amino acid free carboxyl group
$\mathcal{I}^{e}_{aa}$	AAEI	amino acids electrostatic interaction
$\Delta G_{\mathcal{I}^{e}_{aa}}$	AAEIDG	amino acids electrostatic interaction delta G
$\mathcal{I}^{h}_{aa}$	AAHI	amino acids hydrophobic interaction
$\Delta G_{\mathcal{I}^{h}_{aa}}$	AAHIDG	amino acids electrostatic interaction delta G
$\mathcal{B}_{aa}$	AAHB	amino acids hydrogen bonding
$\mathcal{I}X_{aa}$	AAIX (X = 1, 2, 3)	amino acids interaction
$\mathcal{P}_{aa}$	AAP	amino acids pairing
$\Delta G_{\mathcal{P}_{aa}}$	AAPDG	aa pairing delta G
$\mathcal{I}^{e}_{b}$	BEI	bases electrostatic interaction
$\Delta G_{\mathcal{I}^{e}_{b}}$	BEIDG	bases electrostatic interaction delta G
$\mathcal{B}X_{b2}$	BHBX (X = 1, 2, 3)	two bases hydrogen bonding
$\mathcal{I}^{h}_{b}$	BHI	bases hydrophobic interaction
$\Delta G_{\mathcal{I}^{h}_{b}}$	BHIDG	bases hydrophobic interaction delta G
$\mathcal{P}_{b2}$	BP	base pairing
$\Delta G_{\mathcal{P}_{b2}}$	BPDG	base pairing delta G
$\mathcal{F}^{s}$	FS	folding step
$\Delta G_{\mathcal{F}^{s}}$	FSDG	folding step delta G
$\mathcal{I}X_{n}$	NIX (X = 1, 2)	nucleotides interaction
$\mathcal{F}^{s}_{p}$	PFS	protein folding step
$\mathtt{I}_{\mathtt{p}}$	PI	protein inside
$\mathtt{O}_{\mathtt{p}}$	PO	protein outside
$\mathcal{F}^{s}_{rna}$	RNAFS	RNA folding step
$\mathtt{I}_{rna}$	RNAI	RNA inside
$\mathcal{S}$	S	stacking
$\mathcal{B}X_{b3}$	TBHBX (X = 1, 2, 3)	three bases hydrogen bonding
$\mathcal{P}_{b3}$	TBP	triple base pairing
$\Delta G_{\mathcal{P}_{b3}}$	TBPDG	triple base pairing delta G

Table 1.2: Processes of the higher abstraction level

State\Process	Transliteration	Description
$\mathtt{C}$	C	amino acid carboxyl group
$\mathtt{B}_{\mathtt{dr}}$	DR	double-ring base (purine)
$\Delta G_{\mathscr{F}^s}$	FSDG	folding step delta G
$\mathtt{N}$	N	amino acid amino group
$\mathcal{I}_n^h$	NHI	nucleotide hydrophobic interaction
$\Delta G_{\mathcal{I}_n^h}$	NHIDG	nucleotide hydrophobic interaction delta G
$\mathscr{F}_p^s$	PFS	protein folding step
$\mathscr{F}_{rna}^s$	RNAFS	RNA folding step
$\mathtt{O}_{\mathtt{rna}}$	RNAO	RNA outside
$\mathtt{B}_{\mathtt{sr}}$	SR	single-ring base (pyrimidine)
$\mathcal{P}_{aa3}$	TAAP	triple amino acid pairing
$\Delta G_{\mathcal{P}_{aa3}}$	TAAPDG	triple amino acid pairing delta G
$\mathtt{U}_{\mathtt{aa3}}$	TAAU	triple amino acids unit
$\mathtt{U}_{\mathtt{b3}}$	TBU	triple base unit

Table 1.3: Actions

Action	Description
`aa`	amino acid
`aa1fco`	first amino acid free carboxyl group
`aa1fnh`	first amino acid free amino group
`aa2fco`	second amino acid free carboxyl group
`aa2fnh`	second amino acid free amino group
`bb`	buriede bases
`bsc`	buried side chain
`dr`	double-ring base (purine)
`esc`	exposed side chain
`hb`	hydrogen bond
`hbsc`	hydrophobic side chain
hbi	hydrophobic interaction
`hlsc`	hydrophilic side chain
ii	ionic interaction
`ndg`	negative delta G
`paa`	paired amino acids
`pdg`	positive delta G
`sb`	stacked bases
`sr`	single-ring base (pyrimidine)
`tpb`	three paried bases
`ub`	unpaired base
vdwi	van der Waals interaction
`zdg`	zero delta G

Table 1.4: Actions of the higher abstraction level

Action	Description
`bc`	buried component
`ec`	exposed component
hb	hydrogen bonding interaction
`hbc`	hydrophobic component
`hlc`	hydrophilic component
`pu`	paired unit
`tpu`	triple unit
`uu`	unpaired unit

2 Models Construction

In the models of the folding process that we have defined, the weak interactions are classified in three main categories:

- hydrogen bonds;
- electrostatic interactions (ionic and van der Waals);
- hydrophobic interactions.

The hydrogen bond could be defined as an electrostatic interaction, but due to its distinctive properties and the fundamental role it carries out in the folding process, it has been represented separately.

All the weak interactions listed above have been modelled to formally describe the whole folding process. Each folding process always starts from a linear sequence (of nucleotides in RNAs and of amino acids in proteins) and is driven by the reduction in free energy between two different folded configurations.

To better clarify this concept, we can imagine the folding process as a sequence of folding steps, each contributing to the entire process with a new weak interaction between two units of the sequence (equally for RNAs and proteins). In order for a folding step to take place, the weak interaction must cause a reduction in the free energy of the system, which means that the folding step must have a negative ΔG. The ΔG variation during folding is represented as a process that can produce three possible outputs: negative, a positive or zero ΔG.

2.1 Base pairing

In *RNA*, hydrogen bonds allow the pairing between two bases. According to Watson-Crick base pairing, adenine (A) always pairs with uracil (U) with two hydrogen bonds, while guanine (G) always pairs with cytosine (C) with three hydrogen bonds. At the same time, the non-conventional base pairing shows various combinations of the four RNA bases, forming two hydrogen bonds (or even only one); it is not infrequent to find in RNA also a triple base pairing (indeed, it is possible that a unique base quartet forms between G-C base pairs at the junction of two helices).

The hydrogen bond formation (in both Watson-Crick and Wobble base pair) has been modelled generalising that process as an interaction between a purine (adenine or guanine - labelled `dr`, since they are **d**ouble-**r**ing bases) and a pyrimidine (uracil and cytosine - **s**ingle-**r**ing bases and hence labelled `sr`) or between a two paired bases and a third base (also in this case, a generic purine or pyrimidine). The base pairing is symmetric, thus: `srdr` = `drsr`.

To remove some details not necessary for the aim of the model, it has also been opted for another generalisation, not explicitly representing all the possible interaction between a couple of paired bases and a third base, but indicating this process as a "triple base-pairing" ($\mathcal{P}_{b3}$) and its output as "three paired bases" (`tpb`).

For the same reason the formation of the G-C base quartet is not treated in the model.

Regarding the number of hydrogen bonds allowed in a base pair, in our models they must be at least two and at most three; the number of hydrogen bonds that link an unpaired base to a group of two already paired bases must be from one to three. It has been decided to limit the minimum number of hydrogen bonds in a base pair (to the number of two) because base pairs with a single hydrogen bond can be classified as a variant of the primary types and because the whole number of hydrogen bonds found in a base triplet is at least three.

Moreover, because up to now the only known base pair that involves three hydrogen bonds is the one between cytosine and guanine, only the `srdr` base pair is allowed in the model to form a triple hydrogen bond; this means that also AU, GU and CA base pairs could potentially form a triple hydrogen bond, which is a stretch of the current knowledge on hydrogen bonding. Since this property is important for the stability of the RNA molecules, we want to better justify the proposed abstraction: if we want to capture the constraint of limiting the formation of three hydrogen bonds only to the GC base pair, we should represent explicitly every bases and their combination instead of the convention adopted; this would reduce the readability of our models to capture a property that not affect the main purpose for which they were created.

The *Base Pairing* process ($\mathcal{P}_{b2}$) takes two unpaired bases (`ub`) as input and provides as output the two bases paired only if it can form at least two hydrogen bonds (`hb`) between them.

$\mathcal{P}_{b2}$ is a sub-process of a general $\mathcal{F}^s_{rna}$ (*RNA Folding Step*) process, from which it receives its input (the $\mathcal{F}^s_{rna}$ process will be described later in this section); it is one of the possible sub-processes that give to each folding step its specificity. As explained in the article, each folding step, and therefore each base pairing process, is conditioned by the value of the ΔG: it can take place only if its ΔG is negative.

The Triple Base pairing process ($\mathcal{P}_{b3}$) takes as input a couple of bases, paired by the $\mathcal{P}_{b2}$ process, and a third unpaired base (`ub`) and provides as output a group of three paired bases (`tpb`). The number of hydrogen bonds that can be generated in this process is at least one and at most three.

Like the $\mathcal{P}_{b2}$ process, $\mathcal{P}_{b3}$ is a sub-process of $\mathcal{F}^s_{rna}$ and depends on the value of the ΔG (the output of the $\Delta G_{\mathcal{F}^s}$ process) to take place.

The following is the specification of the $\mathcal{P}_{b2}$ and the $\mathcal{P}_{b3}$ processes using Milner's CCS (in the subsection 2.4 on page 8 they will be contextualised in the complete description of the $\mathcal{F}^s_{rna}$ process):

$$\mathcal{P}_{b2} \stackrel{\text{def}}{=} \texttt{hb}.\mathcal{B}1_{b2};$$
$$\mathcal{B}1_{b2} \stackrel{\text{def}}{=} \texttt{hb}.\mathcal{B}2_{b2};$$
$$\mathcal{B}2_{b2} \stackrel{\text{def}}{=} \texttt{hb}.\mathcal{B}3_{b2} + \overline{\texttt{srsr}}.\mathcal{F}^s_{rna} + \overline{\texttt{drdr}}.\mathcal{F}^s_{rna} + \overline{\texttt{srdr}}.\mathcal{F}^s_{rna};$$
$$\mathcal{B}3_{b2} \stackrel{\text{def}}{=} \overline{\texttt{srdr}}.\mathcal{F}^s_{rna};$$

$$\mathcal{P}_{b3} \stackrel{\text{def}}{=} \texttt{hb}.\mathcal{B}1_{b3};$$
$$\mathcal{B}1_{b3} \stackrel{\text{def}}{=} \texttt{hb}.\mathcal{B}2_{b3} + \overline{\texttt{tpb}}.\mathcal{F}^s_{rna};$$
$$\mathcal{B}2_{b3} \stackrel{\text{def}}{=} \texttt{hb}.\mathcal{B}3_{b3} + \overline{\texttt{tpb}}.\mathcal{F}^s_{rna};$$
$$\mathcal{B}3_{b3} \stackrel{\text{def}}{=} \overline{\texttt{tpb}}.\mathcal{F}^s_{rna}.$$

$\mathcal{B}1_{b2}$, $\mathcal{B}2_{b2}$, $\mathcal{B}3_{b2}$ (base hydrogen bond) and $\mathcal{B}1_{b3}$, $\mathcal{B}2_{b3}$, $\mathcal{B}3_{b3}$ (three bases hydrogen bond) are states that allow counting the number of the hydrogen bonds.

In *proteins*, an hydrogen bond can form between the amino group of one amino acid and the carboxyl group of another. Every amino acid has an amino group and a carboxyl group covalently linked to the alpha (central) carbon. In the rest of this document, the terms "amino groups" and "carboxyl groups" will refer specifically to such functional groups. In contrast with the base pairing of nucleotides, only a single hydrogen bond is allowed between two amino acids; however, there is no limitation in the length of a sequence of amino acids linked to one another via hydrogen bonds.

Therefore, two amino acids can hydrogen bond to each other only if they meet the following conditions:

- the interaction has a negative ΔG;
- the amino group of one of the two interacting amino acids and the carboxyl group of the other are both free (not involved in an hydrogen bond).

The *Amino Acids Pairing* process ($\mathcal{P}_{aa}$) is a subprocess of the general $\mathcal{F}^s_p$ (protein folding step), as $\mathcal{P}_{b2}$ is a subprocess of $\mathcal{F}^s_{rna}$.

$\mathcal{P}_{aa}$ takes two amino acids (`aa`) as input, makes an hydrogen bond between the free amino group of the first one (`aa1fnh`) and the free carboxyl group of the second one (`aa2fco`) or between the free carboxyl group of the first amino acids (`aa1fco`) and the free amino group of the second one (`aa2fnh`); then, provides a group of two paired amino acids (`paa` - paired amino acids) as output.

It is important to notice that:

1. although the distinction between "first" and "second" amino acid might appear unnecessary when they are both unpaired, it has to be specified to deal with the situation in which at least one of the two amino acids is already involved in an hydrogen bond through one of its functional groups;
2. when the $\mathcal{P}_{aa}$ process receives two amino acids as input, we have the certainty that an hydrogen bond will form, because the negative ΔG of the interaction has already been checked in the early phases of the $\mathcal{F}^s_p$ process.

The following is the CCS specification of the $\mathcal{P}_{aa}$ process:

$$\mathcal{P}_{aa} \stackrel{\text{def}}{=} \texttt{aa1fnh}.\texttt{NH}_{\texttt{aa1}} + \texttt{aa1fco}.\texttt{CO}_{\texttt{aa1}};$$
$$\texttt{NH}_{\texttt{aa1}} \stackrel{\text{def}}{=} \texttt{aa2fco}.\texttt{CO}_{\texttt{aa2}};$$
$$\texttt{CO}_{\texttt{aa1}} \stackrel{\text{def}}{=} \texttt{aa2fnh}.\texttt{NH}_{\texttt{aa2}};$$
$$\texttt{CO}_{\texttt{aa2}} \stackrel{\text{def}}{=} \texttt{hb}.\mathcal{B}_{aa};$$
$$\texttt{NH}_{\texttt{aa2}} \stackrel{\text{def}}{=} \texttt{hb}.\mathcal{B}_{aa};$$
$$\mathcal{B}_{aa} \stackrel{\text{def}}{=} \overline{\texttt{paa}}.\mathcal{F}^s_p.$$

$\texttt{NH}_{\texttt{aax}}$ and $\texttt{CO}_{\texttt{aax}}$ (where x is 1 or 2) are state that indicate the selection of the free amino group or of the free carboxyl group (respectively) of the x-th amino acid.

2.2 Electrostatic interactions

Two particles electrically charged can interact according to the Coulomb's law. The model of the folding process does not investigate the interactions at atomic level, therefore the details of this law will not be covered. What we need to know is that two elementary units of either an RNA or a protein sequence, can interact in a folding step if they are both charged and if the ΔG of such interaction is negative. The main purpose of this kind of interactions is to stabilise the folded structure reached through the previous steps.

The electrostatic interaction can be of two types: ionic and van der Waals.

The ionic interactions cause the formation of a weak bond between two ions of opposite charge; the van der Waals interactions occur between two molecules oppositely polarised.

The modelling of these interactions is basically the same in both RNA and Protein folding: given as input a couple of bases (in the RNA model) or amino acids (in the Protein model), each unpaired or already paired, the *electrostatic interaction* process allows the nondeterministic choice between a ionic interaction (*ii*) or a van der Waals interaction (*vdwi*), which are produced as output.

The *Bases Electrostatic Interaction* process ($\mathcal{I}^e_b$) specifies the electrostatic interactions in the RNA folding model:

$$\mathcal{I}^e_b \stackrel{\text{def}}{=} \overline{ii}.\mathcal{F}^s_{rna} + \overline{vdwi}.\mathcal{F}^s_{rna}.$$

The *Amino Acids Electrostatic Interaction* process ($\mathcal{I}^e_{aa}$) specifies the electrostatic interactions in the protein folding model:

$$\mathcal{I}^e_{aa} \stackrel{\text{def}}{=} \overline{ii}.\mathcal{F}^s_p + \overline{vdwi}.\mathcal{F}^s_p;$$

$\mathcal{I}^e_b$ is a subprocess of $\mathcal{F}^s_{rna}$; $\mathcal{I}^e_{aa}$ is a subprocess of $\mathcal{F}^s_p$.

2.3 Hydrophobic interactions

Water is a polar solvent, this means that it easily dissolves charged or polar compounds, which are called, for this reason, hydrophilic (from Greek, "water-loving"). In contrast, nonpolar molecules are hydrophobic.

In *RNA*, the purine and pyrimidine bases are hydrophobic and relatively insoluble in water, while the backbone of alternating ribose and phosphate groups is hydrophilic.

To minimize contact of the bases with water and stabilizing the three-dimensional structure of the RNA, during the folding process, the backbone is placed on the outside of the molecule, facing the surrounding water, while the bases are positioned inside, stacked with the planes of their rings parallel to each other (a process called hydrophobic stacking interaction).

In the RNA folding model, the *Bases Hydrophobic Interaction* process ($\mathcal{I}_b^h$) takes two bases as input, produces an hydrophobic interaction for both of them (*hbi*) and provides as output the same bases buried inside the RNA (bb) and stacked to each other (sb).

Since $\mathcal{I}_b^h$ is a subprocess of $\mathcal{F}_{rna}^s$, the fact that the ΔG of the interaction is negative has already been checked in the earlier phases of the latter process.

The CSS specification of the $\mathcal{I}_b^h$ process is:

$$\mathcal{I}_b^h \stackrel{\text{def}}{=} hbi.\mathtt{I}_{rna};$$

$$\mathtt{I}_{rna} \stackrel{\text{def}}{=} \overline{\mathtt{bb}}.\mathcal{S};$$

$$\mathcal{S} \stackrel{\text{def}}{=} \overline{\mathtt{sb}}.\mathcal{F}_{rna}^s.$$

In *proteins*, the specific characteristics of an amino acid are determined by the properties of its R group; the polarity of that group varies widely, from non-polar and hydrophobic to highly polar and hydrophilic. Hydrophobic amino acid side chains tend to be clustered in the protein's interior, away from water, while hydrophilic side chains remain on the protein surface. Folding of a polypeptide chain thus creates an "inside" and an "outside" and generates buried and exposed amino acid side chains. The interior of a protein is generally a densely packed core of hydrophobic amino acid side chains.

The hydrophobic interactions in proteins do not exhibit the stacking phenomenon, therefore the *Amino Acids Hydrophobic Interaction* process ($\mathcal{I}_{aa}^h$) takes only one amino acid as input. Then, if the amino acid side chain is hydrophilic (hlsc), it is exposed outside the protein (esc), if the side chain is hydrophobic (hbsc), it is buried inside the protein (bsc).

The inside and the outside of the protein are identified by the states $\mathtt{I_p}$ and $\mathtt{O_p}$ respectively. $\mathcal{I}_{aa}^h$ is a subprocess of $\mathcal{F}_p^s$.

The following is the CCS specification of the $\mathcal{I}_b^h$ process:

$$\mathcal{I}_{aa}^h \stackrel{\text{def}}{=} \mathtt{hlsc}.\mathtt{O_p} + \mathtt{hbsc}.\mathtt{I_p};$$

$$\mathtt{O_p} \stackrel{\text{def}}{=} \overline{\mathtt{esc}}.\mathcal{F}_p^s;$$

$$\mathtt{I_p} \stackrel{\text{def}}{=} \overline{\mathtt{bsc}}.\mathcal{F}_p^s.$$

2.4 Folding step

Now that we have described the model of each weak interaction in both RNA and protein, it is possible to contextualise these models in the folding step they belong to ($\mathcal{F}_{rna}^s$ or $\mathcal{F}_p^s$) .

Each step represents an iteration which allows the nondeterministic choice of one of the possible weak interaction subprocess.

$\mathcal{F}_{rna}^s$ and $\mathcal{F}_p^s$ ensure that each subprocess complies with the specific restrictions on its input (according to the descriptions made above in this section) and that the interaction has a negative ΔG (and hence can be carried out).

The CCS specification of the whole $\mathcal{F}^s_{rna}$ process is the following:

$$\mathcal{F}^s_{rna} \stackrel{\text{def}}{=} \texttt{ub}.\mathcal{I}1_n + \texttt{ub}.\mathcal{I}2_n + \texttt{srsr}.\mathcal{I}1_n + \texttt{drdr}.\mathcal{I}1_n + \texttt{srdr}.\mathcal{I}1_n + \texttt{tpb}.\mathcal{I}1_n;$$

$$\mathcal{I}1_n \stackrel{\text{def}}{=} \texttt{ub}.\Delta G_{\mathcal{I}^e_b} + \texttt{srsr}.\Delta G_{\mathcal{I}^e_b} + \texttt{drdr}.\Delta G_{\mathcal{I}^e_b} + \texttt{srdr}.\Delta G_{\mathcal{I}^e_b} + \texttt{tpb}.\Delta G_{\mathcal{I}^e_b};$$

$$\mathcal{I}2_n \stackrel{\text{def}}{=} \texttt{ub}.\Delta G_{\mathcal{P}_{b2}} + \texttt{ub}.\Delta G_{\mathcal{I}^h_b} + \texttt{srsr}.\Delta G_{\mathcal{P}_{b3}} + \texttt{drdr}.\Delta G_{\mathcal{P}_{b3}} + \texttt{srdr}.\Delta G_{\mathcal{P}_{b3}};$$

$$\Delta G_{\mathcal{I}^e_b} \stackrel{\text{def}}{=} \texttt{ndg}.\mathcal{I}^e_b;$$

$$\Delta G_{\mathcal{I}^h_b} \stackrel{\text{def}}{=} \texttt{ndg}.\mathcal{I}^h_b;$$

$$\Delta G_{\mathcal{P}_{b2}} \stackrel{\text{def}}{=} \texttt{ndg}.\mathcal{P}_{b2};$$

$$\Delta G_{\mathcal{P}_{b3}} \stackrel{\text{def}}{=} \texttt{ndg}.\mathcal{P}_{b3};$$

$$\mathcal{P}_{b2} \stackrel{\text{def}}{=} \texttt{hb}.\mathcal{B}1_{b2};$$

$$\mathcal{B}1_{b2} \stackrel{\text{def}}{=} \texttt{hb}.\mathcal{B}2_{b2};$$

$$\mathcal{B}2_{b2} \stackrel{\text{def}}{=} \texttt{hb}.\mathcal{B}3_{b2} + \overline{\texttt{srsr}}.\mathcal{F}^s_{rna} + \overline{\texttt{drdr}}.\mathcal{F}^s_{rna} + \overline{\texttt{srdr}}.\mathcal{F}^s_{rna};$$

$$\mathcal{B}3_{b2} \stackrel{\text{def}}{=} \overline{\texttt{srdr}}.\mathcal{F}^s_{rna};$$

$$\mathcal{P}_{b3} \stackrel{\text{def}}{=} \texttt{hb}.\mathcal{B}1_{b3};$$

$$\mathcal{B}1_{b3} \stackrel{\text{def}}{=} \texttt{hb}.\mathcal{B}2_{b3} + \overline{\texttt{tpb}}.\mathcal{F}^s_{rna};$$

$$\mathcal{B}2_{b3} \stackrel{\text{def}}{=} \texttt{hb}.\mathcal{B}3_{b3} + \overline{\texttt{tpb}}.\mathcal{F}^s_{rna};$$

$$\mathcal{B}3_{b3} \stackrel{\text{def}}{=} \overline{\texttt{tpb}}.\mathcal{F}^s_{rna};$$

$$\mathcal{I}^e_b \stackrel{\text{def}}{=} \overline{ii}.\mathcal{F}^s_{rna} + \overline{vdwi}.\mathcal{F}^s_{rna};$$

$$\mathcal{I}^h_b \stackrel{\text{def}}{=} hbi.\texttt{I}_{rna};$$

$$\texttt{I}_{rna} \stackrel{\text{def}}{=} \overline{\texttt{bb}}.\mathcal{S};$$

$$\mathcal{S} \stackrel{\text{def}}{=} \overline{\texttt{sb}}.\mathcal{F}^s_{rna}.$$

$\mathcal{I}1_n$ and $\mathcal{I}2_n$ (nucleotide interaction) are states that allow the selection the right subprocess on the basis of its permitted inputs.

$\Delta G_{\mathcal{P}_{b2}}$ (base pairing delta G), $\Delta G_{\mathcal{P}_{b3}}$ (triple base pairing delta G), $\Delta G_{\mathcal{I}^e_b}$ (bases electrostatic interaction delta G) and $\Delta G_{\mathcal{I}^h_b}$ (bases hydrophobic interaction delta G) processes check that the ΔG of the related interaction is negative.

The CCS specification of the whole $\mathcal{F}_p^s$ (Protein folding step) process is:

$$\mathcal{F}_p^s \stackrel{\text{def}}{=} \texttt{aa}.\mathcal{I}1_{aa} + \texttt{aa}.\Delta G_{\mathcal{I}_{aa}^h};$$

$$\mathcal{I}1_{aa} \stackrel{\text{def}}{=} \texttt{aa}.\Delta G_{\mathcal{I}_{aa}^e} + \texttt{aa}.\Delta G_{\mathcal{P}_{aa}};$$

$$\Delta G_{\mathcal{I}_{aa}^e} \stackrel{\text{def}}{=} \texttt{ndg}.\mathcal{I}_{aa}^e;$$

$$\Delta G_{\mathcal{I}_{aa}^h} \stackrel{\text{def}}{=} \texttt{ndg}.\mathcal{I}_{aa}^h;$$

$$\Delta G_{\mathcal{P}_{aa}} \stackrel{\text{def}}{=} \texttt{ndg}.\mathcal{P}_{aa};$$

$$\mathcal{P}_{aa} \stackrel{\text{def}}{=} \texttt{aa1fnh}.\texttt{NH}_{\texttt{aa1}} + \texttt{aa1fco}.\texttt{CO}_{\texttt{aa1}};$$

$$\texttt{NH}_{\texttt{aa1}} \stackrel{\text{def}}{=} \texttt{aa2fco}.\texttt{CO}_{\texttt{aa2}};$$

$$\texttt{CO}_{\texttt{aa1}} \stackrel{\text{def}}{=} \texttt{aa2fnh}.\texttt{NH}_{\texttt{aa2}};$$

$$\texttt{CO}_{\texttt{aa2}} \stackrel{\text{def}}{=} \texttt{hb}.\mathcal{B}_{aa};$$

$$\texttt{NH}_{\texttt{aa2}} \stackrel{\text{def}}{=} \texttt{hb}.\mathcal{B}_{aa};$$

$$\mathcal{B}_{aa} \stackrel{\text{def}}{=} \overline{\texttt{paa}}.\mathcal{F}_p^s;$$

$$\mathcal{I}_{aa}^e \stackrel{\text{def}}{=} \overline{ii}.\mathcal{F}_p^s + \overline{vdwi}.\mathcal{F}_p^s;$$

$$\mathcal{I}_{aa}^h \stackrel{\text{def}}{=} \texttt{hlsc}.\texttt{O}_{\texttt{p}} + \texttt{hbsc}.\texttt{I}_{\texttt{p}};$$

$$\texttt{O}_{\texttt{p}} \stackrel{\text{def}}{=} \overline{\texttt{ec}}.\mathcal{F}_p^s;$$

$$\texttt{I}_{\texttt{p}} \stackrel{\text{def}}{=} \overline{\texttt{bc}}.\mathcal{F}_p^s.$$

$\mathcal{I}1_{aa}$ is a state that allows the selection of the subprocesses that take two amino acids as input. $\Delta G_{\mathcal{P}_{aa}}$ (amino acids pairing delta G), $\Delta G_{\mathcal{I}_{aa}^e}$ (amino acids electrostatic interaction delta G) and $\Delta G_{\mathcal{I}_{aa}^h}$ (amino acids hydrophobic interaction delta G) processes check that the ΔG of the related interaction is negative.

2.5 RNA folding and protein folding

In order to meet the requirement that each interaction must have a negative ΔG, both the $\mathcal{F}_{rna}^s$ and $\mathcal{F}_p^s$ processes are placed in parallel composition with the $\Delta G_{\mathcal{F}^s}$ (folding step delta G) process, defining in this way the overall folding process ($\mathcal{F}_{rna}$ and $\mathcal{F}_p$ respectively).

$$\Delta G_{\mathcal{F}^s} \stackrel{\text{def}}{=} \overline{\texttt{pdg}}.\Delta G_{\mathcal{F}^s} + \overline{\texttt{ndg}}.\Delta G_{\mathcal{F}^s} + \overline{\texttt{zdg}}.\Delta G_{\mathcal{F}^s};$$

$$\mathcal{F}_{rna} \stackrel{\text{def}}{=} (\mathcal{F}_{rna}^s | \Delta G_{\mathcal{F}^s}) \backslash \{\texttt{ndg}, \texttt{pdg}, \texttt{zdg}\};$$

$$\mathcal{F}_p \stackrel{\text{def}}{=} (\mathcal{F}_p^s | \Delta G_{\mathcal{F}^s}) \backslash \{\texttt{ndg}, \texttt{pdg}, \texttt{zdg}\}.$$

2.6 Model checking

It is possible to verify that the biochemical properties of the folding processes are satisfied by the above-described model. We propose here four examples, expressing some properties as HML formulas and establish if they are satisfied performing the model checking.

1. Two unpaired bases (ub) can form an hydrogen bond (hb) if the ΔG of the interaction is negative (ndg):

   $$\mathcal{F}^s_{rna} \models \langle \mathtt{ub}\rangle\langle \mathtt{ub}\rangle\langle \mathtt{ndg}\rangle\langle \mathtt{hb}\rangle tt;$$

2. with a single hydrogen bond it is not possible to form a base pair (srsr, drdr, srdr):

   $$\mathcal{P}_{b2} \models \langle \mathtt{hb}\rangle([\overline{\mathtt{srsr}}]\mathrm{ff} \text{ and } [\overline{\mathtt{srdr}}]\mathrm{ff} \text{ and } [\overline{\mathtt{drdr}}]ff);$$

3. it is possible to form a group of three paired bases (tpb) with only a single hydrogen bond (between an unpaired base and a group of two already paired bases - srsr in this case); obviously, the ΔG of the interaction must be negative:

   $$\mathcal{F}^s_{rna} \models \langle \mathtt{ub}\rangle\langle \mathtt{srsr}\rangle\langle \mathtt{ndg}\rangle\langle \mathtt{hb}\rangle\langle \overline{\mathtt{tpb}}\rangle tt;$$

4. if an amino acid has an hydrophobic side chain (hbsc), it has to be buried inside (bsc) and not exposed outside (esc) the protein:

   $$\mathcal{F}^s_{p} \models \langle \mathtt{aa}\rangle\langle \mathtt{ndg}\rangle\langle \mathtt{hbsc}\rangle(\langle \overline{\mathtt{bsc}}\rangle \mathrm{tt} \text{ and } [\overline{\mathtt{esc}}]ff);$$

The verification that these formulas are satisfied was made with the aid of the model checking function of the web-based tool CAAL. The results are shown in Figure 1.

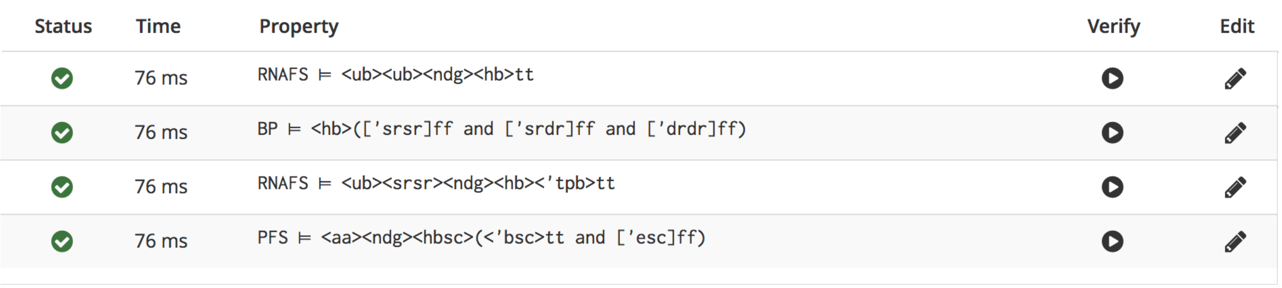

Status	Time	Property	Verify	Edit
✓	76 ms	RNAFS ⊨ <ub><ub><ndg><hb>tt		
✓	76 ms	BP ⊨ <hb>(['srsr]ff and ['srdr]ff and ['drdr]ff)		
✓	76 ms	RNAFS ⊨ <ub><srsr><ndg><hb><'tpb>tt		
✓	76 ms	PFS ⊨ <aa><ndg><hbsc>(<'bsc>tt and ['esc]ff)		

Figure 1. Verification of some biochemical properties, expressed as HML formulas, performed by the CAAL web-based tool. The checkmarks on the "Status" column indicate that all the formulas are satisfied.

2.7 Higher abstraction level model

We might therefore wonder if *there is an abstraction level at which the two folding processes would show a behavioural equivalence*. As it will be proved in this article, this level of abstraction can actually be defined. Its construction, however, requires a generalisation of the weak-interaction processes and the imposition of some limitations to the "expressiveness" of the protein folding process.

The first of the two aforementioned modification can be achieved by:

- redefining nucleotides and the amino acids as general elementary units, which can be paired or unpaired;
- abstracting from the specificity of each pairing process by no longer taking into account the number of hydrogen bonds formed between two (or three) paired units;
- generalising the hydrophobic interactions to their key feature of burying the hydrophobic molecules while exposing the hydrophilic ones (no longer considering the stacking process typical of the hydrophobic interactions of nucleotides).

These adjustments to the model do not affect the main property of each weak interaction, therefore the model is still faithful to the biological process. However they are not sufficient to obtain a behavioural equivalence between the folding processes of RNAs and proteins.

What we still need to do is limiting the folding capability of the proteins by reducing the number of amino acids that can interact through hydrogen bonds to the number of three (the maximum number of nucleotides that can pair in RNAs).

With these considerations in mind, we can rewrite the above model of the folding process.

Base pairing process

$\mathcal{P}_{b2}$ takes two unpaired units (uu) as input (from the $\mathscr{F}^s_{rna}$ process) and produces a paired unit (pu) as output. *The label hb not indicates a single hydrogen bond, but stands for the overall interaction based on hydrogen bonding*.

$$
\begin{aligned}
\mathcal{P}_{b2} &\stackrel{\text{def}}{=} hb.\mathtt{B_{sr}B_{sr}} + hb.\mathtt{B_{dr}B_{dr}} + hb.\mathtt{B_{sr}B_{dr}};\\
\mathtt{B_{sr}B_{sr}} &\stackrel{\text{def}}{=} \overline{\mathtt{pu}}.\mathscr{F}^s_{rna};\\
\mathtt{B_{dr}B_{dr}} &\stackrel{\text{def}}{=} \overline{\mathtt{pu}}.\mathscr{F}^s_{rna};\\
\mathtt{B_{sr}B_{dr}} &\stackrel{\text{def}}{=} \overline{\mathtt{pu}}.\mathscr{F}^s_{rna}.
\end{aligned}
$$

$\mathtt{B_{sr}B_{sr}}$, $\mathtt{B_{dr}B_{dr}}$, $\mathtt{B_{sr}B_{dr}}$ are states that specify the type of base pair of the produced paired unit.

Triple base pairing process

The $\mathcal{P}_{b3}$ process takes an unpaired unit (uu) and a paired unit (pu) as input (from the $\mathscr{F}^s_{rna}$ process) and produces a triple unit (tpu) as output.

$$
\begin{aligned}
\mathcal{P}_{b3} &\stackrel{\text{def}}{=} hb.\mathtt{U_{b3}};\\
\mathtt{U_{b3}} &\stackrel{\text{def}}{=} \overline{\mathtt{tpu}}.\mathscr{F}^s_{rna}.
\end{aligned}
$$

The state $\mathtt{U_{b3}}$ (triple base unit) indicates that an hydrogen bonding interaction (possibly made by more than one hydrogen bond) has taken place.

Amino acid pairing process

The $\mathcal{P}_{aa}$ process takes two unpaired units (uu) as input (from the $\mathscr{F}^s_p$ process) and produces a paired unit (pu) as output. As the same for the base pairing process, *the label hb not indicates a single hydrogen bond*.

$$
\begin{aligned}
\mathcal{P}_{aa} &\stackrel{\text{def}}{=} hb.\mathtt{NC} + hb.\mathtt{CN};\\
\mathtt{NC} &\stackrel{\text{def}}{=} \overline{\mathtt{pu}}.\mathscr{F}^s_p;\\
\mathtt{CN} &\stackrel{\text{def}}{=} \overline{\mathtt{pu}}.\mathscr{F}^s_p.
\end{aligned}
$$

The states NC and CN (where N and C stand for amino group and carboxyl group respectively) allow the preservation of the right complementarity of the hydrogen bond interaction between amino acids.

Triple amino acid pairing process

This is a new process (not present in the previous model); it is necessary to limit the capabilities of amino acids to hydrogen-bond with each other; as for the base pairing, at this level of abstraction at most three amino acids can be connected by the same hydrogen bonding interaction (not to be confused with a single hydrogen bond).

The $\mathcal{P}_{aa3}$ process takes an unpaired unit (uu) and a paired unit as input and produces a triple unit (tpu) as output.

$$\mathcal{P}_{aa3} \stackrel{\text{def}}{=} hb.\mathtt{U}_{\mathtt{aa3}};$$
$$\mathtt{U}_{\mathtt{aa3}} \stackrel{\text{def}}{=} \overline{\mathtt{tpu}}.\mathscr{F}^{s}_{p}.$$

Electrostatic interaction

The base electrostatic interaction ($\mathcal{I}^{e}_{b}$) and the amino acid electrostatic interaction ($\mathcal{I}^{e}_{aa}$) processes are unchanged compared with the previous model (see Section 2.2 on page 7).

Nucleotide hydrophobic interaction

Since the hydrophobic stacking is no longer considered in the new model, the hydrophobic interaction can affect a single nucleotide per iteration (folding step).

The process, renamed $\mathcal{I}^{h}_{n}$, takes one unpaired unit as input and buries inside the RNA its hydrophobic component ($\mathtt{hbc}-\mathtt{bc}$) while exposes outside the RNA its hydrophilic component ($\mathtt{hlc}-\mathtt{ec}$).

$$\mathcal{I}^{h}_{n} \stackrel{\text{def}}{=} \mathtt{hlc}.\mathtt{O}_{\mathtt{rna}} + \mathtt{hbc}.\mathtt{I}_{rna}$$
$$\mathtt{O}_{\mathtt{rna}} \stackrel{\text{def}}{=} \overline{\mathtt{ec}}.\mathscr{F}^{s}_{rna};$$
$$\mathtt{I}_{rna} \stackrel{\text{def}}{=} \overline{\mathtt{bc}}.\mathscr{F}^{s}_{rna}.$$

Amino acid hydrophobic interaction

The $\mathcal{I}^{h}_{aa}$ process takes one unpaired unit as input and buries inside the protein its hydrophobic component ($\mathtt{hbc}-\mathtt{bc}$) while exposes outside the protein its hydrophilic component ($\mathtt{hlc}-\mathtt{ec}$). In this case the “component” is a generalisation of the side chain, this means that each unpaired unit taken as input can have an hydrophobic or an hydrophilic component (but not both).

$$\mathcal{I}^{h}_{aa} \stackrel{\text{def}}{=} \mathtt{hlc}.\mathtt{O}_{\mathtt{p}} + \mathtt{hbc}.\mathtt{I}_{\mathtt{p}};$$
$$\mathtt{O}_{\mathtt{p}} \stackrel{\text{def}}{=} \overline{\mathtt{ec}}.\mathscr{F}^{s}_{p};$$
$$\mathtt{I}_{\mathtt{p}} \stackrel{\text{def}}{=} \overline{\mathtt{bc}}.\mathscr{F}^{s}_{p}.$$

Folding step

The $\mathscr{F}^{s}_{rna}$ and $\mathscr{F}^{s}_{p}$ perform the same tasks as in the previous model (see Section 2.4 on page 8).

The CCS specification of the whole modified $\mathscr{F}^s_{rna}$ process is:

$$
\begin{aligned}
\mathscr{F}^s_{rna} &\stackrel{\text{def}}{=} \mathtt{uu}.\mathcal{I}1_n + \mathtt{pu}.\mathcal{I}1_n + \mathtt{uu}.\Delta G_{\mathcal{I}^h_n} + \mathtt{uu}.\mathcal{I}2_n + \mathtt{tpu}.\mathcal{I}1_n;\\
\mathcal{I}1_n &\stackrel{\text{def}}{=} \mathtt{uu}.\Delta G_{\mathcal{I}^e_b} + \mathtt{pu}.\Delta G_{\mathcal{I}^e_b} + \mathtt{tpu}.\Delta G_{\mathcal{I}^e_b};\\
\mathcal{I}2_n &\stackrel{\text{def}}{=} \mathtt{uu}.\Delta G_{\mathcal{P}_{b2}} + \mathtt{pu}.\Delta G_{\mathcal{P}_{b3}};\\
\Delta G_{\mathcal{I}^e_b} &\stackrel{\text{def}}{=} \mathtt{ndg}.\mathcal{I}^e_b;\\
\Delta G_{\mathcal{I}^h_n} &\stackrel{\text{def}}{=} \mathtt{ndg}.\mathcal{I}^h_n;\\
\Delta G_{\mathcal{P}_{b2}} &\stackrel{\text{def}}{=} \mathtt{ndg}.\mathcal{P}_{b2};\\
\Delta G_{\mathcal{P}_{b3}} &\stackrel{\text{def}}{=} \mathtt{ndg}.\mathcal{P}_{b3};
\end{aligned}
$$

$$
\begin{aligned}
\mathcal{P}_{b2} &\stackrel{\text{def}}{=} hb.\mathtt{B_{sr}B_{sr}} + hb.\mathtt{B_{dr}B_{dr}} + hb.\mathtt{B_{sr}B_{dr}};\\
\mathtt{B_{sr}B_{sr}} &\stackrel{\text{def}}{=} \overline{\mathtt{pu}}.\mathscr{F}^s_{rna};\\
\mathtt{B_{dr}B_{dr}} &\stackrel{\text{def}}{=} \overline{\mathtt{pu}}.\mathscr{F}^s_{rna};\\
\mathtt{B_{sr}B_{dr}} &\stackrel{\text{def}}{=} \overline{\mathtt{pu}}.\mathscr{F}^s_{rna};
\end{aligned}
$$

$$
\begin{aligned}
\mathcal{P}_{aa3} &\stackrel{\text{def}}{=} hb.\mathtt{U_{aa3}};\\
\mathtt{U_{aa3}} &\stackrel{\text{def}}{=} \overline{\mathtt{tpu}}.\mathscr{F}^s_p;
\end{aligned}
$$

$$
\begin{aligned}
\mathcal{I}^e_b &\stackrel{\text{def}}{=} \overline{ii}.\mathscr{F}^s_{rna} + \overline{vdwi}.\mathscr{F}^s_{rna};\\
\mathcal{I}^h_n &\stackrel{\text{def}}{=} \mathtt{hlc}.\mathtt{O_{rna}} + \mathtt{hbc}.\mathtt{I}_{rna};\\
\mathtt{O_{rna}} &\stackrel{\text{def}}{=} \overline{\mathtt{ec}}.\mathscr{F}^s_{rna};\\
\mathtt{I}_{rna} &\stackrel{\text{def}}{=} \overline{\mathtt{bc}}.\mathscr{F}^s_{rna}.
\end{aligned}
$$

$\mathcal{I}1_n$ and $\mathcal{I}2_n$ (nucleotide interaction) are states that allow the selection the right subprocess on the basis of its permitted inputs.

$\Delta G_{\mathcal{P}_{b2}}$ (base pairing delta G), $\Delta G_{\mathcal{P}_{b3}}$ (triple base pairing delta G), $\Delta G_{\mathcal{I}^e_b}$ (bases electrostatic interaction delta G) and $\Delta G_{\mathcal{I}^h_n}$ (nucleotide hydrophobic interaction delta G) processes check if the ΔG of the related interaction is negative.

The CCS specification of the whole modified $\mathscr{F}_p^s$ process is the following:

$$
\begin{aligned}
\mathscr{F}_p^s &\stackrel{\text{def}}{=} \texttt{uu}.\mathcal{I}1_{aa} + \texttt{pu}.\mathcal{I}1_{aa} + \texttt{uu}.\Delta G_{\mathcal{I}^h_{aa}} + \texttt{uu}.\mathcal{I}2_{aa} + \texttt{tpu}.\mathcal{I}1_{aa};\\
\mathcal{I}1_{aa} &\stackrel{\text{def}}{=} \texttt{uu}.\Delta G_{\mathcal{I}^e_{aa}} + \texttt{pu}.\Delta G_{\mathcal{I}^e_{aa}} + \texttt{tpu}.\Delta G_{\mathcal{I}^e_{aa}};\\
\mathcal{I}2_{aa} &\stackrel{\text{def}}{=} \texttt{uu}.\Delta G_{\mathcal{P}_{aa}} + \texttt{pu}.\Delta G_{\mathcal{P}_{aa3}};\\
\Delta G_{\mathcal{I}^e_{aa}} &\stackrel{\text{def}}{=} \texttt{ndg}.\mathcal{I}^e_{aa};\\
\Delta G_{\mathcal{I}^h_{aa}} &\stackrel{\text{def}}{=} \texttt{ndg}.\mathcal{I}^h_{aa};\\
\Delta G_{\mathcal{P}_{aa}} &\stackrel{\text{def}}{=} \texttt{ndg}.\mathcal{P}_{aa};\\
\Delta G_{\mathcal{P}_{aa3}} &\stackrel{\text{def}}{=} \texttt{ndg}.\mathcal{P}_{aa3};
\end{aligned}
$$

$$
\begin{aligned}
\mathcal{P}_{aa} &\stackrel{\text{def}}{=} hb.\texttt{NC} + hb.\texttt{CN};\\
\texttt{NC} &\stackrel{\text{def}}{=} \overline{\texttt{pu}}.\mathscr{F}_p^s;\\
\texttt{CN} &\stackrel{\text{def}}{=} \overline{\texttt{pu}}.\mathscr{F}_p^s;
\end{aligned}
$$

$$
\begin{aligned}
\mathcal{P}_{aa3} &\stackrel{\text{def}}{=} hb.\texttt{U}_{\texttt{aa3}};\\
\texttt{U}_{\texttt{aa3}} &\stackrel{\text{def}}{=} \overline{\texttt{tpu}}.\mathscr{F}_p^s;
\end{aligned}
$$

$$
\begin{aligned}
\mathcal{I}^e_{aa} &\stackrel{\text{def}}{=} \overline{ii}.\mathscr{F}_p^s + \overline{vdwi}.\mathscr{F}_p^s;\\
\mathcal{I}^h_{aa} &\stackrel{\text{def}}{=} \texttt{hlc}.\texttt{O}_\texttt{p} + \texttt{hbc}.\texttt{I}_\texttt{p};\\
\texttt{O}_\texttt{p} &\stackrel{\text{def}}{=} \overline{\texttt{ec}}.\mathscr{F}_p^s;\\
\texttt{I}_\texttt{p} &\stackrel{\text{def}}{=} \overline{\texttt{bc}}.\mathscr{F}_p^s.
\end{aligned}
$$

$\mathcal{I}1_{aa}$ and $\mathcal{I}2_{aa}$ are states that allow the selection of the right subprocess on the basis of its permitted inputs. $\Delta G_{\mathcal{P}_{aa}}$ (amino acids pairing delta G), $\Delta G_{\mathcal{P}_{aa3}}$ (triple amino acids pairing delta G), $\Delta G_{\mathcal{I}^e_{aa}}$ (amino acids electrostatic interaction delta G) and $\Delta G_{\mathcal{I}^h_{aa}}$ (amino acids hydrophobic interaction delta G) processes check if the ΔG of the related interaction is negative.

The folding processes are still defined as the parallel composition of the folding step process and the folding step ΔG (see Section 2.5 on page 10).
